# Parent-offspring regression to estimate the heritability of an HIV-1 trait in a realistic setup

**DOI:** 10.1186/s12977-017-0356-3

**Published:** 2017-05-23

**Authors:** Nadine Bachmann, Teja Turk, Claus Kadelka, Alex Marzel, Mohaned Shilaih, Jürg Böni, Vincent Aubert, Thomas Klimkait, Gabriel E. Leventhal, Huldrych F. Günthard, Roger Kouyos, the Swiss HIV Cohort Study

**Affiliations:** 1 Department of Infectious Diseases and Hospital Epidemiology, University Hospital Zurich, Zurich, Switzerland; 2 Institute of Medical Virology, University of Zurich, Zurich, Switzerland; 3 Divisions of Immunology and Allergy, University Hospital Lausanne, Lausanne, Switzerland; 4 Molecular Virology, Department Biomedicine - Petersplatz, University of Basel, Basel, Switzerland; 5 Department of Environmental Systems Science, ETH Zurich, Zurich, Switzerland; 6 Department of Civil and Environmental Engineering, Massachusetts Institute of Technology (MIT), Cambridge, USA

**Keywords:** Heritability, Parent-offspring regression, HIV-1, Set-point viral load, Ornstein–Uhlenbeck process, Mixed-effect model

## Abstract

**Background:**

Parent-offspring (PO) regression is a central tool to determine the heritability of phenotypic traits; i.e., the relative extent to which those traits are controlled by genetic factors. The applicability of PO regression to viral traits is unclear because the direction of viral transmission—who is the donor (parent) and who is the recipient (offspring)—is typically unknown and viral phylogenies are sparsely sampled.

**Methods:**

We assessed the applicability of PO regression in a realistic setting using Ornstein–Uhlenbeck simulated data on phylogenies built from 11,442 Swiss HIV Cohort Study (SHCS) partial pol sequences and set-point viral load (SPVL) data from 3293 patients.

**Results:**

We found that the misidentification of donor and recipient plays a minor role in estimating heritability and showed that sparse sampling does not influence the mean heritability estimated by PO regression. A mixed-effect model approach yielded the same heritability as PO regression but could be extended to clusters of size greater than 2 and allowed for the correction of confounding effects. Finally, we used both methods to estimate SPVL heritability in the SHCS. We employed a wide range of transmission pair criteria to measure heritability and found a strong dependence of the heritability estimates to these criteria. For the most conservative genetic distance criteria, for which heritability estimates are conceptually expected to be closest to true heritability, we found estimates ranging from 32 to 46% across different bootstrap criteria. For less conservative distance criteria, we found estimates ranging down to 8%. All estimates did not change substantially after adjusting for host-demographic factors in the mixed-effect model (±2%).

**Conclusions:**

For conservative transmission pair criteria, both PO regression and mixed-effect models are flexible and robust tools to estimate the contribution of viral genetic effects to viral traits under real-world settings. Overall, we find a strong effect of viral genetics on SPVL that is not confounded by host demographics.

**Electronic supplementary material:**

The online version of this article (doi:10.1186/s12977-017-0356-3) contains supplementary material, which is available to authorized users.

## Background

A key question in viral infections is to what extent viral genetic factors contribute to infection traits. A relevant illustrative example for this question is the large variability in HIV-1 set-point viral load (SPVL) [1] and the associated effect of SPVL on the progression time to AIDS [2]. Several studies have previously estimated the heritability of SPVL using different methods, resulting in highly variable estimates ranging from 6 to 51% [3–9, 15].

This wide range of estimates can only partly be attributed to differences in datasets, as the choice of heritability estimation method also plays a crucial role [10]. “True” (broad-sense) heritability is defined as the fraction of trait variance explained by genetic factors [11]. Parent-offspring (PO) regression, the focus of our analysis, provides estimates that are conceptually closest to this definition. PO regression is the traditional quantitative genetics tool to measure heritability. PO regression compares trait values in parents to trait values in their offspring and uses the slope of the linear regression line of parent (or donor) and offspring (or recipient) trait values as an estimate for heritability. In the special case of a population consisting of only parent-offspring pairs with identical viral genomes, the slope of the PO regression yields by definition the heritability of the trait [11]. Because ideally no or little viral evolution occurs between parent and offspring, the heritability estimates from PO regression are insensitive to different models of trait evolution. By contrast, phylogenetic comparative methods fit specific models of trait evolution to the trait on the transmission tree; examples of these methods include Pagel’s lambda [12], phylogenetic mixed models [13, 14] and a recent improvement of this method [15]. It is unclear how well such a model can reflect the complex process of evolution of an HIV-1 trait like set-point viral load. Indeed, Leventhal and Bonhoeffer [10] recently showed in a simulation study using Wright-Fisher populations that PO regression yielded better heritability estimates and claimed that as long as model assumptions of phylogenetic methods could not be adjusted to higher adequacy, PO regression should be preferred over estimates from phylogenetic methods. Thereby they supported earlier results that suggested using PO regression as an unbiased estimator of SPVL heritability [5].

Nevertheless, in a realistic setup transmission pairs generally do not have an identical genotype, and the heritability estimate thus depends on the transmission pair definition. PO regression also neglects the trait information of patients that are not in transmission pairs; a large dataset is therefore needed in order to maintain adequate statistical power. Two key challenges arise when applying PO regression in a clinical setting: Firstly, when extracting transmission pairs from phylogenetic trees, it is typically impossible to identify directionality, i.e., who is donor and recipient [16, 17]. Secondly, due to sparse sampling, transmission pairs detected on the phylogeny are generally not “real” transmission pairs, but merely represent the closest patients in the transmission chain with available viral sequence data.

Here, we assess the applicability of PO regression in a realistic clinical setting using sequence and viral load data from the Swiss HIV Cohort Study (SHCS). In particular, using simulated data on phylogenies derived from SHCS sequence data, we test PO regression robustness against both the lack of knowledge concerning transmission directionality and sparse sampling. We then consider clinical SPVL data and show, using a mixed-effect model variant of PO-regression, that the observed heritability is not due to confounding host demographic factors.

## Results

### Study population

We assessed the suitability of PO regression using simulated traits and thereafter estimated heritability of SPVL data on the SHCS transmission network. 11,442 of the 19,227 individuals enrolled in the SHCS had available viral sequence data (March 3, 2016) and were hence included in the phylogenetic analysis. The number of transmission pairs extracted from these phylogenies (and included in the PO regression) depended on the genetic distance and support-value criteria used for defining these pairs. We considered a range of such criteria because, intuitively, we expected a trade-off between accuracy of the PO regression and statistical power: On the one hand, PO regression between pairs with zero viral genetic distance yields the heritability of a trait by definition [5]. Hence, the accuracy of estimates should increase with decreasing genetic distance, arguing for strict distance criteria. On the other hand, only small number of pairs will fit very strict criteria. For the full SHCS phylogeny, with genetic distance thresholds of 0.005, 0.01, 0.02, 0.03 substitutions/site and no condition on bootstrap values, the number of extracted transmission pairs was 471, 918, 1605 and 2023, respectively. Using a minimum bootstrap value of 0.7 (0.9), the respective numbers of detected transmission pairs decreased to 403, 781, 1372, 1696 (291, 525, 849, 990).

### Random donor and recipient identification

**Fig. 1:**
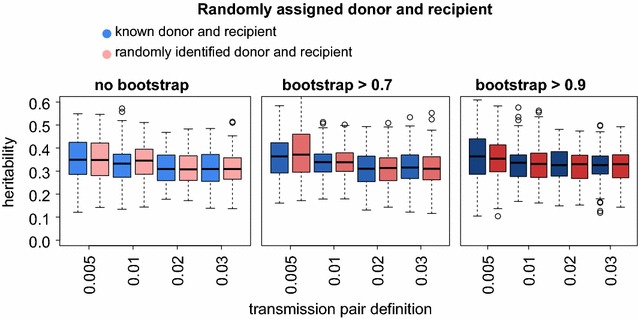
Randomly assigned donor and recipient. For each of 12 transmission pair criteria (combination of allowed genetic distance and bootstrap cutoff), heritability measurements of 100 runs of OU-simulated trait values were obtained using only the 178 transmission pairs for which there was strong evidence for the directionality of transmission [18]. For each of the criteria, the PO estimates using known versus randomly assigned donors and recipients are compared. In each *boxplot* the *black line* near the *middle* of the *box* is the median value of the group. The *top* and *bottom* of the *box* represent the 25th and 75th percentile of the data and the *vertical size* of the *box* is therefore the interquartile range, or IQR. The “whisker”, or the *arrows* extending out of the *box*, show the reasonable extremes of the data, which we took as 1.5 × IQR (as it is the default in R). The individual points represent outliers

As it is typically impossible to identify who is donor and recipient in viral transmission pairs (i.e., the direction of transmission), we assessed the hypothesis that random donor and recipient assignment does not influence the heritability estimates of PO regression. In a previous study, the directionality of transmission was assessed for a subset of 202 phylogenetically identified transmission pairs of the SHCS with estimated seroconversion dates. This enabled drawing a conclusion on the directionality of transmission with high confidence [18]. Of these, 178 transmission pairs (with <3% genetic distance and no bootstrap) occurred in our tree as well. Based on these specific pairs and 100 Ornstein–Uhlenbeck (OU) simulated trait values for them, we compared the heritability estimates derived by PO regression when using the “true” donors and recipients to randomly assigning donors and recipients. These simulations exhibited no difference between heritability estimates based on known and randomly chosen donors and recipients (Fig. 1), indicating that potential misidentification of donors and recipients does not affect our heritability measurements.

### Sparse sampling

**Fig. 2:**
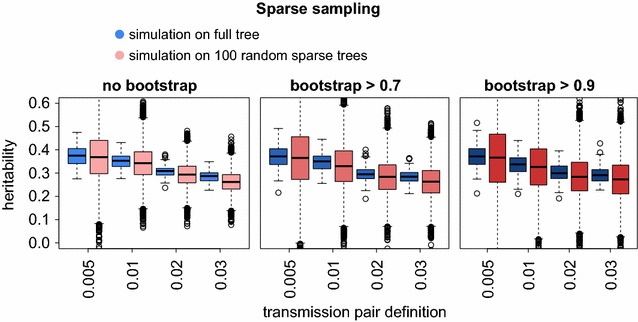
Sparse sampling. For each of 12 transmission pair criteria (combination of allowed genetic distance and bootstrap cutoff) the heritability was estimated using PO regression on the full SHCS phylogeny (*blue bars*) and on 100 randomly generated sparse trees with sparseness 1/3 (*red bars*). For both estimations, the same 100 realizations of OU simulations on the full SHCS phylogeny and the 100 sparse phylogenies were used

We next addressed the question of how sparse sampling influences the reliability of PO heritability estimates. Sequence and trait data is usually only available for a subset of the population under consideration. Hence, transmission pairs identified in the phylogeny will often not represent actual pairs, but rather just the closest sequences sampled. To study the impact of sparse sampling on PO regression, we constructed 100 sample phylogenetic trees, each with one-third of the sequences of the full tree (for the impact of other sparseness levels, see Additional file 1: Figure S3). We performed 100 OU simulations on the full tree. For each of these simulations and each transmission pair criterion, we determined the heritability using transmission pairs from the full tree and from the 100 sparse trees. We observed that PO regression is robust to sparse sampling, in the sense that median heritability estimates are only weakly affected by sparse sampling, with the differences being particularly small when transmission pairs were extracted according to a conservative definition (Fig. 2). As expected however, variation around the median increases considerably in sparse trees (Additional file 1: Figure S3), which may make heritability estimation in sparsely sampled populations less reliable.

### Dependence on transmission pair criterion

**Fig. 3:**
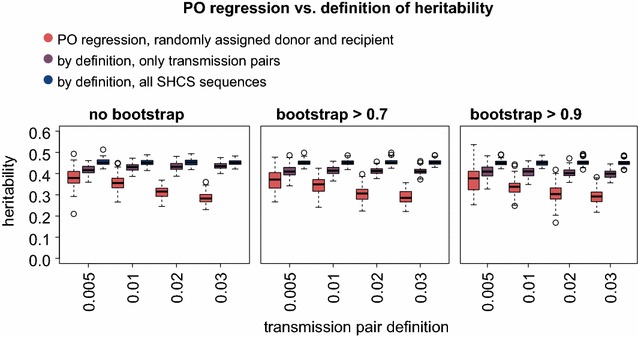
PO regression versus definition of heritability. For each of 12 transmission pair criteria (combination of allowed genetic distance and bootstrap cutoff), three heritability definitions are compared: PO regression with randomly assigned donor and recipient, the true heritability (variance of genetic component over the overall variance) applied only to the transmission pairs that were included in the PO regression and the original definition applied to all SHCS tips of the tree. The *boxplots* represent heritability measurements from 100 realizations of the OU process

From a theoretical perspective, it is clear that PO regression performs perfectly if viral genetic distance between transmission pairs is zero. However, in a realistic setting this scenario is not feasible—therefore, we addressed the impact of genetic distance in the transmission pair criterion on the PO estimator. Generally, we found that the heritability estimated by parent-offspring regression provides a lower bound for the true heritability. The extent to which PO regression underestimates the true heritability depends both on the distance-cutoff used to define pairs and on the strength of selection implemented in the OU process: For the standard version of the OU process used here (corresponding to the maximum likelihood fit of OU to our data), we find that PO regression provides a close estimate for the strictest distance criteria (Fig. 3). For weaker selection parameters (which are however still in the large highest posterior density intervals), we find that PO regression provides close estimates for a much broader range of transmission pair criteria (Additional file 2: Figure S2). This indicates that accuracy of PO regression depends on the transmission pair criteria and that this dependence increases with the strength of directional selection.

Additionally, we aimed to understand the influence of neglecting tips of our phylogeny that are not members of transmission pairs on our heritability estimates. Heritability estimates were higher when measured on all patients of the phylogeny versus only on transmission pairs, with a more pronounced effect for stricter criteria (Fig. 3). This is due to a non-random selection of the tips belonging to transmission pairs. Since the transmission pairs belong to a specific subpopulation, this reiterates that heritability is a trait of a population because the measured heritability depends on both the expressed variation of a trait in a population as well as the variation in population specific environmental components.

### Comparison with mixed effect model

**Fig. 4:**
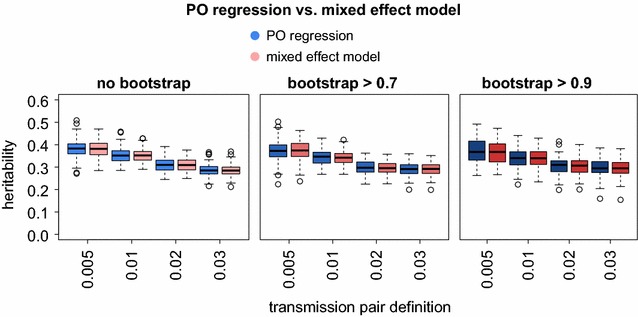
PO regression versus mixed effect model. For each of 12 transmission pair criteria (combination of allowed genetic distance and bootstrap cutoff), the heritability estimates of PO regression and mixed effect models are compared using 100 realizations of an OU process. For the mixed effect model no covariates were included in order to allow direct comparison to PO regression

An alternative approach to measure heritability on pairs is to use a linear mixed effect model, in which the pairs are viewed as independent groups. In this case, heritability corresponds to the ratio of the between-group variance to the total variance. Both methods, PO regression and mixed effect models yielded nearly identical heritability estimates (Fig. 4). This is in line with the theoretical expectation that the two approaches are equivalent. The mixed effect approach has however two advantages: (1) it can be naturally extended to clusters of more than two members (important when considering transmission clusters), (2) it allows to adjust the effect for potential confounders by including them as covariates in the model. This is not possible in PO regression since including a potential confounder as a covariate in the linear regression would only account for its effect on the “offspring” but not for its effect on the “parent”. We next explored these advantages on real clinical data using HIV-1 SPVL as a case study.

### Sensitivity analysis

The above OU-based simulation analyses employed the maximum likelihood model parameter estimates for HIV-1 SPVL values using the POUMM R package [15]. In a sensitivity analysis (Additional file 3: Figure S1), we find that PO regression provides reliable heritability estimates over a broad range of parameters of the OU model (Additional file 4).

### PO regression to measure SPVL heritability

**Fig. 5:**
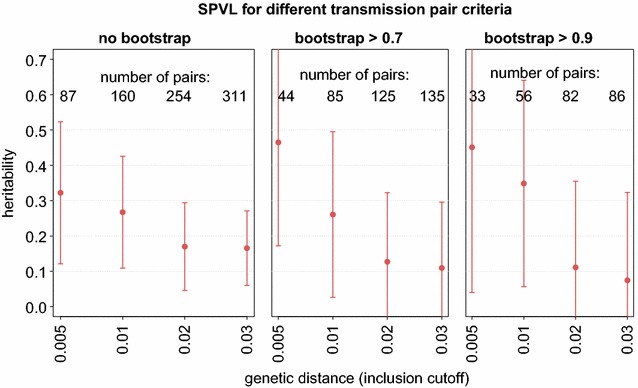
SPVL heritability for different transmission pair criteria. On the sparse tree that includes only sequences of patients with available SPVL, we measured heritability of SPVL using the same 12 criteria as were employed in the simulated data (combination of allowed genetic distance and bootstrap cutoff). For the intuition of the tradeoff between statistical power and methodological correctness, also 95% confidence intervals and the number of transmission pairs included in the analysis are shown

In order to estimate the heritability of SPVL in the SHCS, we identify transmission pairs as cherries (adjacent tips with a common ancestor node) on the phylogeny that was constructed from patients with available SPVL and assign donor and recipients randomly. This approach of considering only the 29% of patients with SPVL and random PO assignment is justified by the results of our simulations. With this approach we find between 45 and 380 pairs for the 12 different pair criteria and a SPVL heritability of 8–46%, respectively (Fig. 5). Notably, we observe a considerably higher heritability for pairs that are defined by stricter distance criteria, for example 32–46% across different bootstrap values for transmission pairs with a genetic distance smaller than 0.5%. We suggest that the heritability estimates derived with these conservative definitions are closest to the true heritability, even though they are associated with low sample sizes and large statistical error (see “Discussion” section). The SPVL results are qualitatively in line with our simulation results, but the effect of distance is more pronounced for the real SPVL data than for the simulated data.

### The influence of covariates on the heritability estimate

**Fig. 6:**
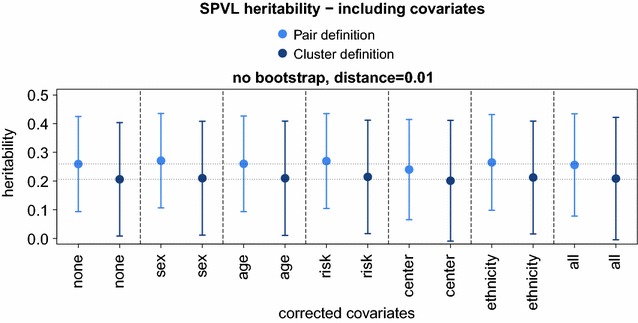
SPVL heritability—including covariates. Heritability was measured using a mixed-effect model on transmission pairs and transmission clusters using the same definition of genetic distance <0.01 and bootstrap >0. We corrected for the host factors sex, age, risk (MSM, HET, IDU), ethnicity and center of treatment, separately (*middle bars*) and altogether (*rightest bars*)

Confounding effects can influence heritability estimates. Our simulations show that mixed effect models estimate heritability equally well as PO regression. Importantly, mixed effects models can both correct for confounders and include transmission clusters of size greater than two, leading to an increase in statistical power. We simultaneously tested five possible confounding effects—gender, age, transmission route (MSM, heterosexual, IDU), ethnicity and center of treatment—and found no significant influence on the heritability estimate (Fig. 6). On the other hand, an inclusion of clusters of size greater than two lowered the estimate. One possible interpretation of this is the increase of the average evolutionary distance through the inclusion of further patients within the distance cutoff compared to transmission pairs.

## Discussion

 Our results reiterate that the heritability of a trait is a property of the population in which it is measured; even the “true” (broad-sense) heritability will differ for different subpopulations. Using transmission pairs of a phylogeny, strictly speaking, one therefore cannot infer the heritability of a trait on the whole phylogeny, but only the heritability of the trait in the “transmission pair subpopulation”. The fact that heritability in the transmission pair population differ from the overall population and PO estimates depend strongly on the transmission pair criteria puts into perspective the strongly varying SPVL heritability estimates in the literature [3–8, 15]. We conclude that differences in SPVL heritability estimates between different cohorts, as well as between entire cohorts and specific subpopulations, are to be expected; for example extremely high heritability for MSM in the SHCS [3]. Nevertheless, our conceptually most credible PO regression-based estimates of set-point viral load heritability measured with conservative transmission pair criteria (32–46%) are in the upper range of previous estimates [3–8, 15].

On the one hand side, for strict transmission pair criteria, our heritability estimates are in the upper range of previous results. This might be because we only consider set-point viral loads from individuals with a low within-patient variability of chronic-phase viral load measurements. By contrast, most other studies consider the first viral load measurement regardless of the subsequent fluctuation of this quantity. This implies that these studies might have underestimated heritability by considering individuals that do not have a well-defined SPVL and thereby misidentified measurement noise as an environmental contribution to that trait. In accordance with this interpretation and similarly to [3], we also find considerably lower heritability estimates (10% lower on average) when including also more fluctuating SPVL measurements and such with unknown fluctuation (Additional file 5: Figure S4).

On the other hand, for liberal genetic distance cutoffs we gain statistical power but at the cost of methodological correctness: Pairs with large genetic distance violate the assumption of “no or little evolution” between transmission pairs. As it is this assumption which intuitively justifies the use of transmission pairs for parent-offspring regression, including such pairs may lead to underestimates of heritability (Fig. 5) and potentially sensitivity of the estimates from PO regression to different models of trait evolution could arise.

A limitation of our study is given by the quite large range of plausible alpha and sigma parameters for our OU simulations. However, we are confident that the maximum likelihood estimate we chose for our simulations represents the fitted data the best (considering the decrease in heritability estimate with liberal criteria) and verified that our results on misidentification of donor and recipient, and imperfect sampling are not sensitive to the change of these parameters within the range of the large highest posterior density intervals.

An additional limitation of PO regression in general is given by a potential selection towards transmission pairs with recent transmission, which we cannot account for due to many missing infection dates. However, we are considering a trait independent of infection time and do therefore not expect confounding of our heritability estimates.

## Conclusion

Our study provides a validation of the applicability and robustness of PO regression to estimate heritability of viral traits in a realistic (i.e., imperfect) clinical setting using the SHCS as a case study. We simulated data based on a phylogeny constructed of viral sequence data and showed that two potential challenges—misidentification of donor and recipient, and imperfect sampling—do not systematically distort heritability estimates by PO regression.

From the example of SPVL in the SHCS we learn that the heritability estimates depend on the employed transmission pair selection criteria, with higher heritability estimates for stricter criteria. Despite lower statistical power, we suggest that conservative criteria should be preferred, because, conceptually, true heritability is estimated at zero genetic distance, i.e., in the case where parent and offspring have identical genomes. This also represents a key limitation of PO regression and vice versa an advantage of phylogenetic comparative methods: only big datasets contain enough conservatively defined transmission pairs to draw statistically.

Finally, we showed that a mixed-effect model with pairs as groups yielded heritability estimates equivalent to PO regression. This model has the advantage of being able to also take into account clusters with more than two members and to correct for the effect of covariates. Applying this model to SPVL showed that the heritability estimates of SPVL are robust to adjusting for demographic factors, suggesting that the high heritability of SPVL is not due to the clustering of such factors on the viral phylogeny. Additionally, considering clusters of size >2 led to lower SPVL estimates, which could be due to the increase of the average evolutionary distance through the inclusion of further patients within the distance cutoff compared to transmission pairs.

We conclude that PO regression is for conservative transmission pair criteria a robust estimator of heritability (and provides a lower bound for these with liberal transmission pair criteria) in large datasets and that its conceptual problems are not quantitatively relevant in a real-life setting.

## Methods

### The Swiss HIV Cohort Study

The Swiss HIV Cohort Study (SHCS) is a large prospective, multi-center study established in 1988 [19] including a drug resistance database that contains HIV sequences for approximately 60% (11,442) of all patients enrolled (>19,200). Since 1996 the SHCS drug resistance database covers even 75% of all patients because more than 11,000 genotypes were performed on plasma samples derived from the SHCS biobank. The sequence database was checked for potential duplicates of the same patients if distances were zero or very small and errors were fixed together with the datacentre. At each semi-annual follow-up visit, laboratory and clinical data are obtained. The SHCS includes HIV-infected individuals aged ≥18 years and is highly representative of the HIV epidemic in Switzerland, covering at least an estimated 45% of total HIV infections reported to the Swiss health authorities [19] including some hard-to-reach populations [20]. Written informed consent was obtained for each SHCS study participant.

### Set-point viral load data

Our set-point viral load (SPVL) measurements were based on the log10 RNA measurements more than 180 days after the first evidence of an HIV infection (given by either the (1) earliest documented positive HIV test, (2) the self-reported positive HIV test or (3) the SHCS registration date in absence of (1) and (2)), while the CD4 count was >300, the patient was untreated and there were no AIDS symptoms. Using this definition ensures that the patient is within the asymptomatic phase of the HIV infection with generally stable viral load. For 5698 SHCS patients there was at least one RNA value available within this range. However, we found that the within-patient RNA variability is large in some patients: several RNA values fall in the range of possible set-point viral load values [21]. Therefore, we added the restriction that more than one RNA value per patient needed to be available and that the variability among those values had to be smaller than 0.4 log10 copies/ml. This restriction decreased the number of individuals in the SHCS with SPVL values to 3293 (see Additional file 5: Figure S4 for a sensitivity analysis regarding the effect of this restriction).

### Phylogenetic tree construction

11,442 partial pol sequences from SHCS cohort participants (one per patient) and 19,252 blasted Los Alamos background sequences were pooled together and aligned to an HXB2 reference genome using MUSCLE [22]. Insertions relative to HXB2 and resistance mutations according to the Stanford (http://hivdb.stanford.edu/) and International Antiviral Society-USA (https://www.iasusa.org/) lists were removed [18], and positions with many gaps were eliminated by Gblocks [23]. Conserved blocks from multiple alignments were selected for phylogenetic analysis and a phylogenetic tree was reconstructed with FastTree using a generalized time-reversible model [24]. One hundred Bootstrap trees were constructed using FSEQBOOT, and bootstrap values were assigned to the original tree using the script *CompareToBootstrap.pl* of the FastTree package. All sparse trees in our analysis were derived from the original tree using the “drop.tip” function of the R package APE [25]. We chose a sparsity level of one-third since this approximately represents the fraction of SHCS patients on our phylogeny with available SPVL data. A sensitivity analysis was performed to assess the effect of different sparsity levels (Additional file 4).

### Extraction of Transmission pairs

We identified potential transmission pairs using the R package APE [25] and custom scripts as those cherries that fulfilled two criteria: (1) a genetic distance of at least 0.5, 1, 2, and 3%, respectively; and (2) no bootstrap criterion, or a bootstrap of more than 70 or 90%. Since there is no general consensus on what defines a “true” transmission pair [26] from a phylogeny alone, we aimed to understand the effect of various transmission pair definitions on the heritability estimates.

### Simulated traits

To study PO regression, we simulated HIV-1 traits on the phylogenetic tree using the R package APE [25]. An Ornstein–Uhlenbeck (OU) process, which is a generalization of a Brownian motion (BM) that accounts for stabilizing selection, simulates the evolution of the trait on the tree. In the BM model a trait is assumed to evolve according to the stochastic process $\sigma dW_{t}$, where (*W*_*t*_)_(*t*≥0)_ denotes Brownian motion and accounts for randomness in the divergence of a trait, and *σ* scales the magnitude of fluctuations. In the OU model, (*X*_*t*_)_(*t*≥0)_ is defined as

$$dX_{t} = \alpha \left( {\theta - X_{t} } \right)dt + \sigma dW_{t} ;$$

the global optimum level *θ* and the strength of selection *α* can be defined additionally [27]. To account for environmental influences on the trait, a normally distributed term was added to the trait values, after they were generated by the OU model. Suitable parameters for the simulations were inferred from the “well-defined” SHCS SPVL values with restricted variability using a maximum likelihood fit with the R package POUMM [15]. We performed a sensitivity analysis for the four inferred parameters and also describe the uncertainty that remains when simultaneously varying alpha and sigma in the Additional file 4 (Additional file 3: Figure S1; Additional file 2: Figure S2).

### Heritability estimates

Transmission pairs were extracted according to the different criteria described above and donor and recipient were randomly assigned. Then, a linear regression of donor trait values against their respective recipient trait values was performed and the slope of this regression was used as the PO heritability estimator.

For the mixed effect model approach we fitted the trait data with a mixed-effect model (using the R package NLME [28]) with pairs or clusters as grouping variables and determined heritability as the ratio of the between-group variance and the total variance.

## Additional files

**Additional file 1: Figure S3.** Heritability estimates for different sparsity levels. OU simulations were run 100 times on the full SHCS tree and transmission pairs according to the 0.01 distance and 0 bootstrap criterion were extracted. Heritability was then estimated using the PO regression. Next, transmission pairs were extracted on 10 previously built random sparse trees for each sparseness level from 10%-90% and heritability estimates were collected using the same OU realizations.

**Additional file 2: Figure S2.** Simultaneous variation of the OU parameters sigma and alpha. For each of 4 transmission pair criteria (no bootstrap cutoff) in combination with three different alpha and sigma values for the OU simulations, three heritability definitions are compared: PO regression with randomly assigned donor and recipient, the true heritability (variance of genetic component over the overall variance) just applied to the transmission pairs that were included in the PO regression and the original definition applied to all SHCS tips of the tree. The boxplots represent heritability measurements from 100 realizations of the OU process.

**Additional file 3: Figure S1.** Sensitivity analysis. For this figure different parameters for the OU process were compared in light of the goodness of the PO estimator. Standard deviation of the normal distribution and all OU parameters (around the one inferred with ml.poumm function of the POUMM package [15]) were used to simulate a trait 100 times for each parameter combination, for each of them the PO estimator was plotted against the true heritability of only the transmission pairs. The different parameters have the following influence on the simulation: Sd-env: standard deviation of the environmental component (normal distribution). Sigma: standard-deviation of the random component for each branch (constant). Alpha: strength of the selective constraint for each branch (constant). Theta: optimum for each branch (constant).

**Additional file 4.** Supplementary Material.

**Additional file 5: Figure S4.** Sensitivity to SPVL variability cutoff. Using PO regression and a fixed bootstrap cutoff of 0.7, SPVL heritability was estimated for each of the distance criteria and different levels of allowed within patient variability in the SPVL estimates. 95% confidence intervals are shown to demonstrate the decreasing statistical power with increasing (more conservative) variability cutoff.
