# Supplementary figures and images for: Parent-offspring regression to estimate the heritability of an HIV-1 trait in a realistic setup
Source: Retrovirology. 2017 May 23;14:33. doi: 10.1186/s12977-017-0356-3 (PMC5442860; doi:10.1186/s12977-017-0356-3)

# Heritability estimates for different sparseness levels

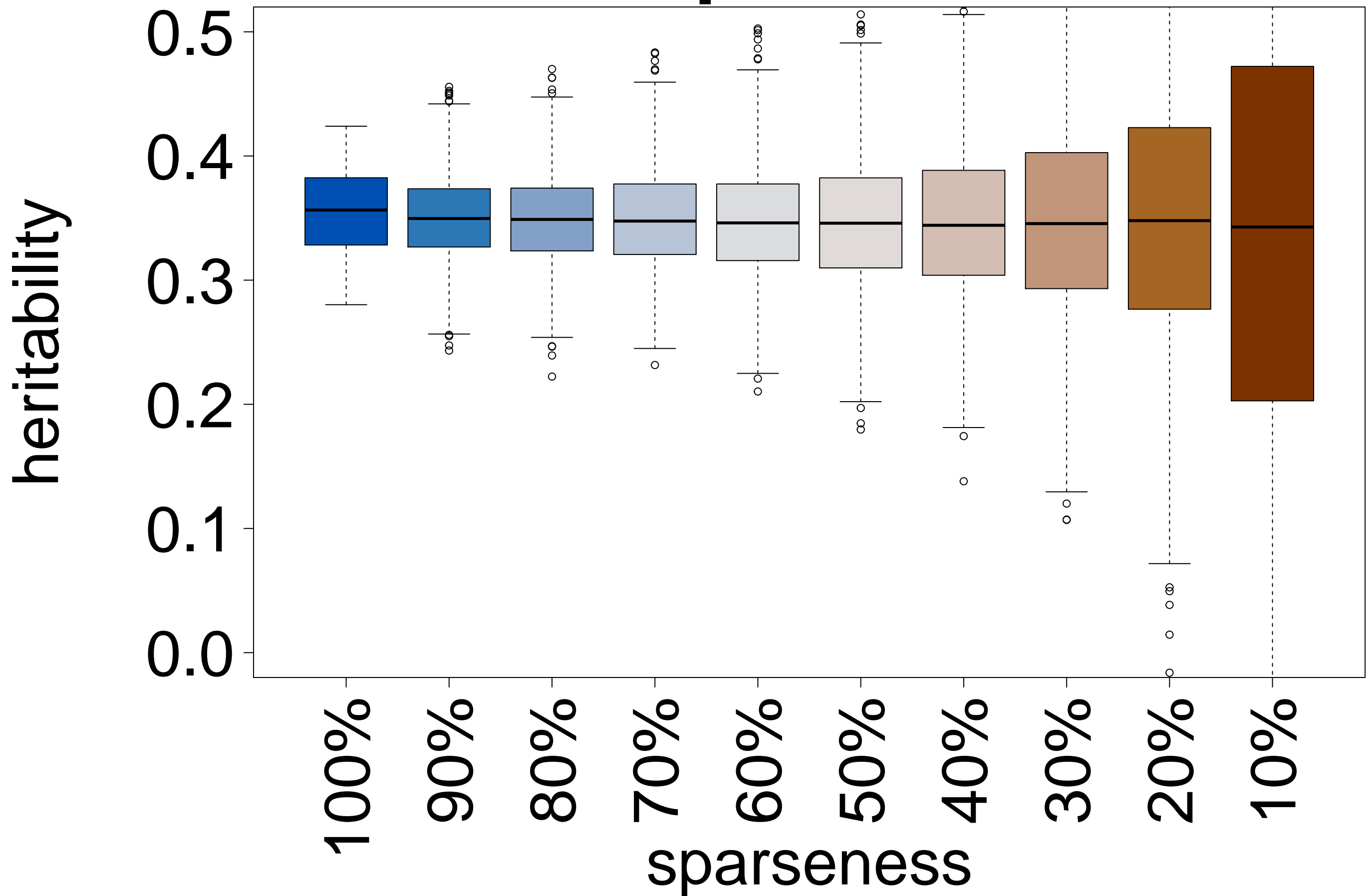

Supplement: Supplementary file 1 — Additional file 1: Figure S3. Heritability estimates for different sparsity levels. OU simulations were run 100 times on the full SHCS tree and transmission pairs according to the 0.01 distance and 0 bootstrap criterion were extracted. Heritability was then estimated using the PO regression. Next, transmission pairs were extracted on 10 previously built random sparse trees for each sparseness level from 10%-90% and heritability estimates were collected using the same OU realizations. [file 12977_2017_356_MOESM1_ESM.pdf]

# Sensitivity analysis

Pair definition: 0 bootstrap, 0.005 distance

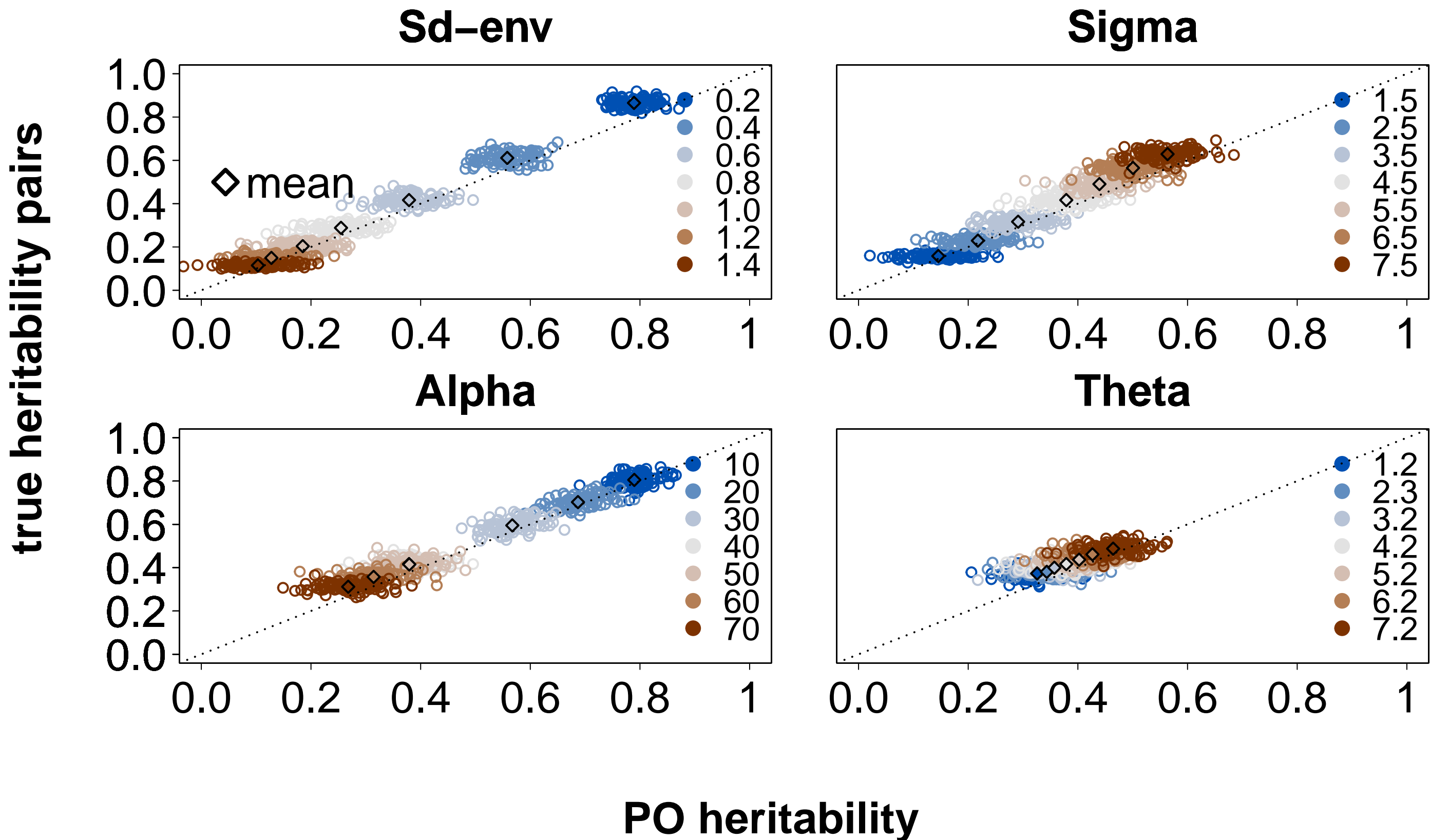

Supplement: Supplementary file 3 — Additional file 3: Figure S1. Sensitivity analysis. For this figure different parameters for the OU process were compared in light of the goodness of the PO estimator. Standard deviation of the normal distribution and all OU parameters (around the one inferred with ml.poumm function of the POUMM package [15]) were used to simulate a trait 100 times for each parameter combination, for each of them the PO estimator was plotted against the true heritability of only the transmission pairs. The different parameters have the following influence on the simulation: Sd-env: standard deviation of the environmental component (normal distribution). Sigma: standard-deviation of the random component for each branch (constant). Alpha: strength of the selective constraint for each branch (constant). Theta: optimum for each branch (constant). [file 12977_2017_356_MOESM3_ESM.pdf]

# Sensitivity to SPVL variability cutoff

Bootstrap > 0.7

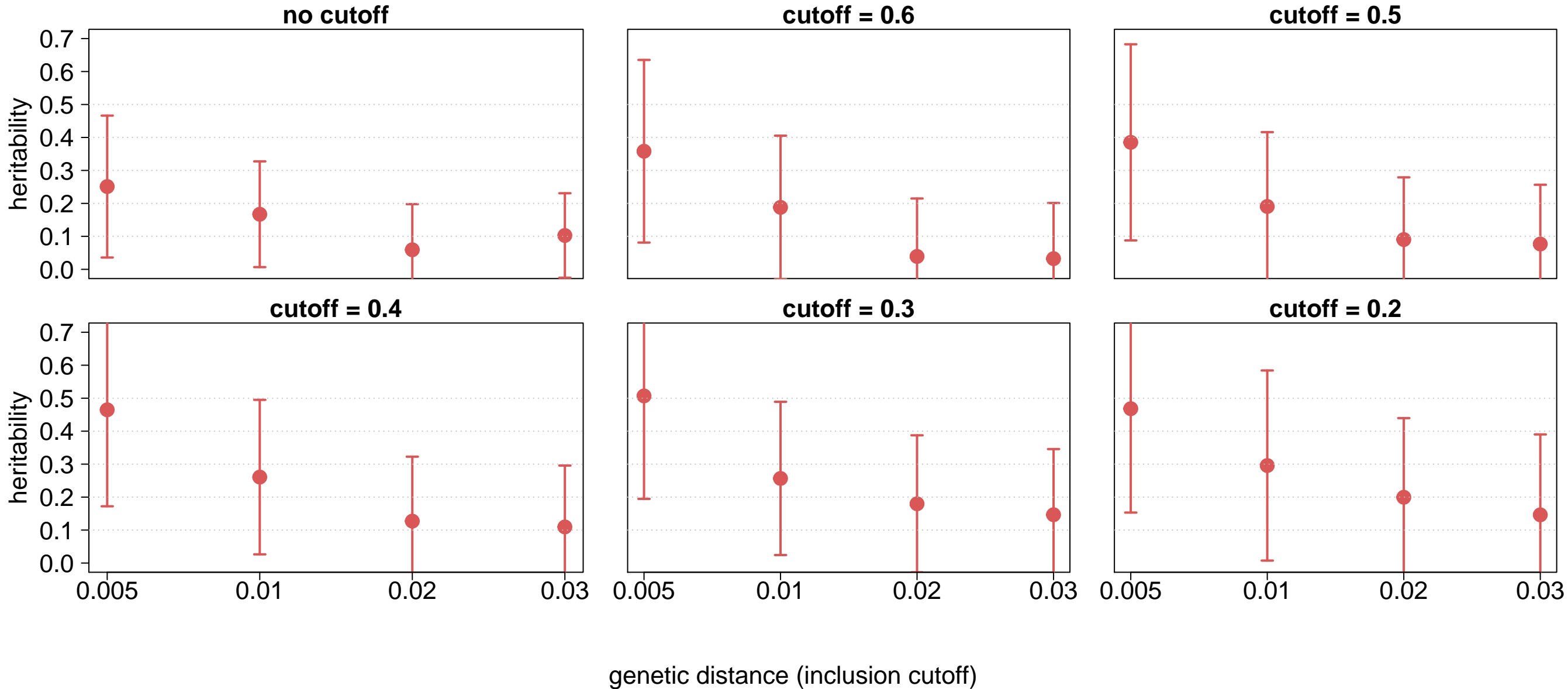

Supplement: Supplementary file 5 — Additional file 5: Figure S4. Sensitivity to SPVL variability cutoff. Using PO regression and a fixed bootstrap cutoff of 0.7, SPVL heritability was estimated for each of the distance criteria and different levels of allowed within patient variability in the SPVL estimates. 95% confidence intervals are shown to demonstrate the decreasing statistical power with increasing (more conservative) variability cutoff. [file 12977_2017_356_MOESM5_ESM.pdf]
