# Supplementary material for: Parent-offspring regression to estimate the heritability of an HIV-1 trait in a realistic setup
Source: Retrovirology. 2017 May 23;14:33. doi: 10.1186/s12977-017-0356-3 (PMC5442860; doi:10.1186/s12977-017-0356-3)
Supplement: Supplementary file 2 — Additional file 2: Figure S2. Simultaneous variation of the OU parameters sigma and alpha. For each of 4 transmission pair criteria (no bootstrap cutoff) in combination with three different alpha and sigma values for the OU simulations, three heritability definitions are compared: PO regression with randomly assigned donor and recipient, the true heritability (variance of genetic component over the overall variance) just applied to the transmission pairs that were included in the PO regression and the original definition applied to all SHCS tips of the tree. The boxplots represent heritability measurements from 100 realizations of the OU process. [file 12977_2017_356_MOESM2_ESM.pdf]

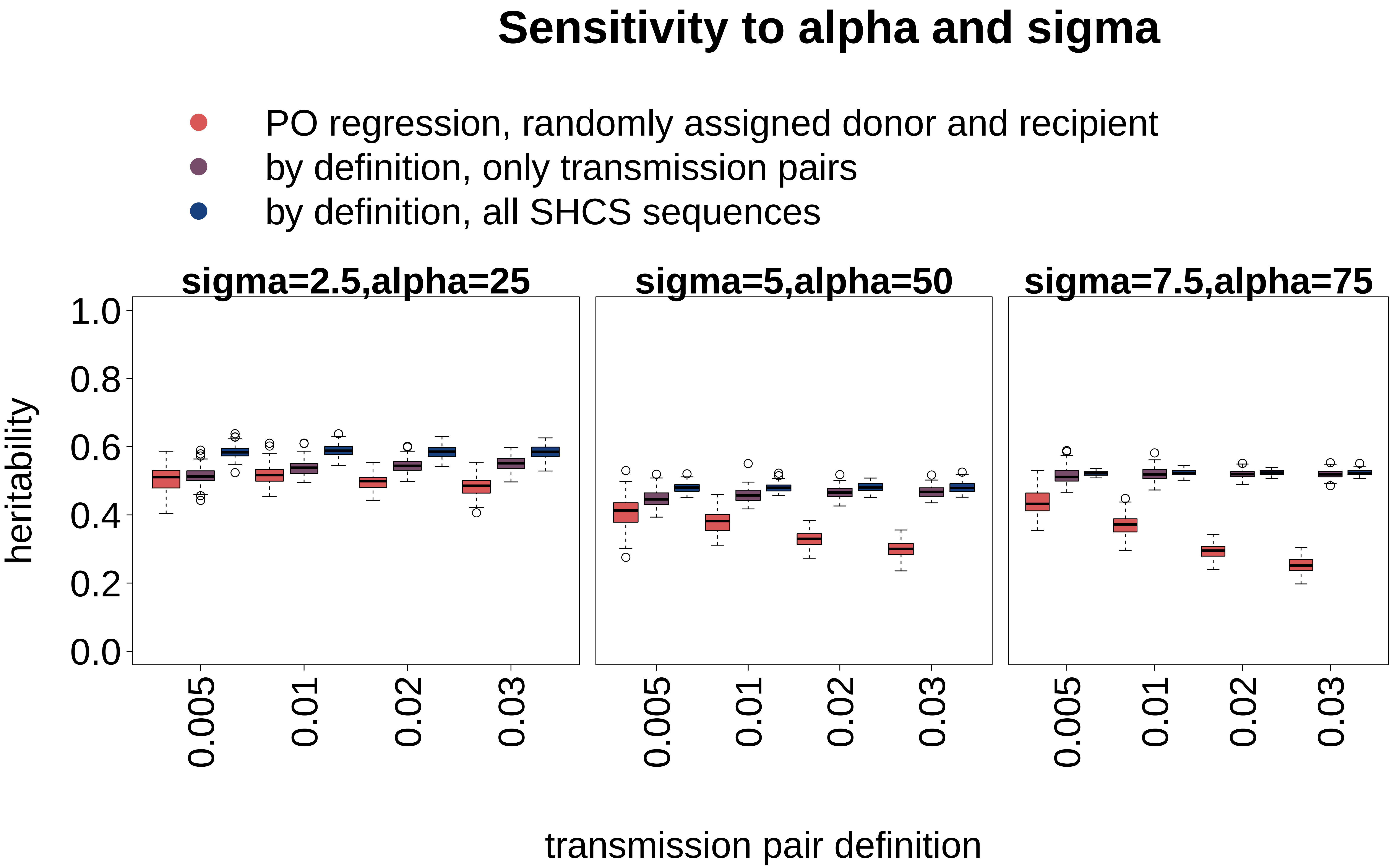

Sensitivity to alpha and sigma
PO regression, randomly assigned donor and recipient
by definition, only transmission pairs
by definition, all SHCS sequences
sigma=2.5,alpha=25
sigma=5,alpha=50
sigma=7.5,alpha=75
heritability
1.0
0.8
0.6
0.4
0.2
0.0
0.005
0.01
0.02
0.03
transmission pair definition
