# Supplementary material for: Parent-offspring regression to estimate the heritability of an HIV-1 trait in a realistic setup
Source: Retrovirology. 2017 May 23;14:33. doi: 10.1186/s12977-017-0356-3 (PMC5442860; doi:10.1186/s12977-017-0356-3)
Supplement: Supplementary file 4 — Additional file 4. Supplementary Material. [file 12977_2017_356_MOESM4_ESM.pdf]

Supplementary Material

A sensitivity analysis was performed to verify that uncertainty in the model parameters do not manipulate the goodness-of-fit of the PO estimate. For each of the parameters—environmental standard deviation and sigma, alpha, theta of the OU process—simulations were run using the parameters suggested by the POUMM package (Mitov and Stadler 2016), as well as six parameter combinations around the recommended values. We showed that for this range of parameters the PO estimates are well in line with the true heritability of the transmission pair population or slightly underestimating the heritability. There is no indication that this should change for other parameters (Figure S1) but one should keep in mind that the underestimation of heritability is expected to grow with more liberal transmission pair criteria.

However, we observed that the OU fit to the SPVL data is sensitive to smaller changes in the data, i.e. adding/removing the restriction to the SPVL. Due to a strong association of alpha and sigma (Pearson correlation > 0.85) we have an identifiability problem, which is also represented by the large highest posterior density (HPD) intervals for alpha and sigma calculated by the POUMM package. Even though we showed that varying the parameters at once does not disturb the heritability estimates, when varying sigma and alpha as much as possible within the HPD intervals, we find results that could allow for different biological interpretations (Figure S2). For a low strength of selection (i.e. alpha low), we observe that PO heritability estimates are almost constant over the different transmission pairs criteria, while for a higher strength of selection the heritability estimates are strongly negatively associated with the distance in the transmission pair criterion. We observe that the parameters, that were actually chosen by the package (sigma=4.6, alpha=50) represent a strength of selection, where the heritability estimates depend on the distance criteria. This is in line with what we observe in the SHCS SPVL heritability estimates, and therefore consider trustworthy.

Another sensitivity analysis was done to describe the impact of different sparsity levels on the PO estimation. For our main analysis we chose a sparsity level of one third since this approximately represents the fraction of SHCS patients on our phylogeny with SPVL data

available. In contrast to our main analysis, this sensitivity analysis was only performed for pairs with distances < 0.01 and bootstrap values > 0. For each of the sparsity levels 10%, 20%, 30%, 40%, 50%, 60%, 70%, 80%, and 90%, 10 random sparse trees were built, prior to the analysis. We ran our trait simulations 100 times on the full SHCS phylogeny and derived a PO heritability value for each of the 100 runs. Then, we also obtained the PO estimate for each OU realization for each of the 10 sparse trees per sparsity level, meaning 1,000 heritability values per sparsity level. We observed that the heritability estimates are qualitatively consistent, but the confidence intervals grow with increasing sparsity, as intuitively expected (Figure S4).

Finally, we also checked the sensitivity of different SPVL variability thresholds and found that the heritability measurement is strongly affected by adding a cutoff (increase of heritability estimate by 10% on average for a threshold of 0.4). This issue could have potentially lowered results of previous studies (Figure S4).
